# *Notes from the Field*: Response to a Case of Travel-Associated Lassa Fever — Iowa, October–November 2024

**DOI:** 10.15585/mmwr.mm7411a3

**Published:** 2025-04-03

**Authors:** Diana L. Von Stein, Alexandra Barger, Andrew Hennenfent, Robert Ramaekers, Amanda Mandi, Kenzie Teno, Karen Brust, Jonathan Simmons, Nicholas Mohr, Lisa Veach, Sudhir Kumar, Aneesa Afroze, Emily McCutchen, Amanda Bartling, Michael Pentella, Megan Nelson, Jennifer Craft, Rikki Hetzler, Amy Thoreson, Alicia Coppedge, Sam Jarvis, Jennifer Miller, Alison M. Todres, Jessica L. Wickline, Sheena Tarrant, Leanna Sayyad, Inna Krapiunaya, Amy Schuh, Amy Whitesell, Gerard C. Kuotu, Kiara McNamara, Nancy Cornish, Shelly Schwedhelm, Angela Vasa, Angela Hewlett, Shantyl Galloway, Aaron D. Kofman, Katrin S. Sadigh, Robert Kruse, Barbara Knust, Matthew Donahue

**Affiliations:** ^1^Division of Public Health, Iowa Department of Health and Human Services; ^2^Epidemic Intelligence Service, CDC; ^3^Kentucky Department for Public Health; ^4^University of Iowa Healthcare, Iowa City, Iowa; ^5^UnityPoint Health, Des Moines, Iowa; ^6^MercyOne Health, Des Moines, Iowa; ^7^Nebraska Public Health Laboratory, Omaha, Nebraska; ^8^State Hygienic Laboratory at the University of Iowa, Coralville, Iowa; ^9^Trinity Muscatine Public Health, Muscatine, Iowa; ^10^Scott County Health Department, Davenport, Iowa; ^11^Johnson County Public Health, Iowa City, Iowa; ^12^National Center for Emerging and Zoonotic Infectious Diseases, CDC; ^13^Laboratory Leadership Service, CDC; ^14^National Center for Immunization and Respiratory Diseases, CDC; ^15^Office of the Director, CDC; ^16^University of Nebraska Medical Center, Omaha, Nebraska.

**Summary**

**What is already known about this topic?**

Lassa fever is a viral hemorrhagic fever (VHF) endemic to western Africa. Before 2024, eight travel-associated cases had been identified in the United States.

**What is added by this report?**

A fatal Lassa fever case in a patient returning from Liberia, the first U.S. case diagnosed in eight years and the ninth U.S. travel-associated case since 1969, was identified in Iowa in late 2024. Investigation identified 180 contacts. Lassa fever virus testing was performed for five symptomatic contacts; all laboratory results were negative.

**What are the implications for public health practice?**

The coordinated public health response to this case underscores the importance of eliciting a travel history from febrile patients, effective VHF planning within public health departments and medical communities, and the importance of rapid local testing capabilities.

## Introduction

Lassa fever is a viral hemorrhagic fever (VHF) endemic to western Africa. The Lassa virus has a *Mastomys* genus rodent reservoir and is transmitted through contact with excreta or body fluids of infected rodents or humans (*1*); the incubation period is 1–3 weeks.* No licensed vaccine to prevent Lassa fever is currently available. In late October 2024, the Iowa Department of Health and Human Services (HHS) received a telephone call from a local hospital regarding a patient who had recently returned from rural construction-related work in Liberia, where Lassa fever is endemic. The patient’s travel history, negative malaria test result, and increasing daily body temperature and hemodynamic instability, despite receipt of empiric broad-spectrum antibiotics, prompted concern for Lassa fever. A blood specimen collected at hospital C was tested within hours at the Nebraska Public Health Laboratory using the BioFire Global Fever Special Pathogens Panel (*2*), which returned a presumptive positive result for Lassa virus. The diagnosis was subsequently confirmed by CDC’s Viral Special Pathogens Branch. This represents the first U.S. Lassa fever case in eight years and the ninth U.S. travel-associated Lassa fever case since 1969 (*3*). An investigation was undertaken by federal, state, and local partners to identify contacts of the patient who might have been exposed and to prevent further transmission.

## Timeline, Investigation, and Outcomes

The patient returned to Iowa from Liberia during early October 2024 (day 0), and experienced fever, myalgias, and headache on day 8. After an evaluation in the emergency department of hospital A on day 15, the patient was transferred to hospital B for diagnostic evaluation. On day 19, the patient needed specialized care and was transferred to hospital C, the hospital that contacted the Iowa Department of HHS; the patient died on day 21.

The patient’s clinical status by the time the diagnosis was recognized at hospital C precluded obtaining detailed previous exposure history. Risk assessments^†^ were completed for 180 contacts (Table). Because illness began >1 week after returning from Liberia, the patient was not considered to have been infectious while in Liberia or during travel from Liberia to Iowa (*4*).

**TABLE:** Exposure group and risk classifications* of contacts of a patient with travel-associated Lassa fever (N = 180) — Iowa, October–November 2024

Exposure location (no.)	Contact risk level (no.)					Total contacts (180)
	High exposure, nature of risk			Low exposure, nature of risk	No exposure	
	Household^†^ (4)	Health care setting with ≥1 PPE omission^†,§,¶^ (95)	Health care setting with ≥1 PPE omission^§,¶^ and skin-to-skin contact or cleaning of body fluids^†^ (6)	No PPE omission^§,¶^ (health care setting) or risk for exposure was possible (community-associated)^†^ (50)	None (25)	
Household (4)	4**	—	—	—	—	**4**
Community (4)	—	3**	—	1	—	**4**
**Health care (172)**						
Hospital A (ED)	—	6	2^††^	1	—	**9**
EMS unit A	—	2	—	—	—	**2**
EMS unit B	—	2	—	—	—	**2**
EMS unit C	—	—	—	—	3	**3**
Hospital B	—	35	3^††^	7	6	**51**
Hospital C^§§^	—	24^††^	1^††^	36	7	**68**
**Postmortem care**	—	—	—	—	3	**3**
**Laboratory**						
A (ED)	—	2^††^	—	—	—	**2**
B	—	13^††^	—	1	1	**15**
C	—	4^††^	—	—	—	**4**
D	—	1^††^	—	1	—	**2**
E	—	3^††^	—	3	2	**8**
F	—	—	—	—	3^†^	**3**

**Abbreviations**: ED = emergency department; EMS = emergency medical services; PPE = personal protective equipment.

* https://www.cdc.gov/viral-hemorrhagic-fevers/php/public-health-strategy/people-with-suspected-or-confirmed-vhf-or-high-risk.html

^†^ Actively monitored for signs and symptoms, including weakness or fatigue, fever, headache, chills, muscles aches, diarrhea, vomiting, unexplained bleeding or bruising, rash, chest pain, sore throat, cough, or dark urine.

^§^ https://www.cdc.gov/viral-hemorrhagic-fevers/hcp/guidance/ppe-clinically-stable-puis.html

^¶^ https://www.cdc.gov/viral-hemorrhagic-fevers/hcp/guidance/ppe-clinically-unstable.html

** Quarantined until 21 days after the last exposure.

^††^ Excluded from work until 21 days after last exposure.

^§§^ The patient was clinically stable until arrival at hospital C.

Among the 180 contacts, four household contacts (2%) and three of the four community-associated contacts (2%) were classified as having high-risk exposures, quarantined until day 21 after their last exposure (the maximum incubation period), and monitored twice daily for Lassa fever signs and symptoms.^§^ Contacts’ monitoring results were submitted by local public health and health care facilities to a REDCap database.

Among the 180 contacts, 172 (96%) were health care–associated, and level of risk was determined by use of personal protective equipment (PPE)^¶^ in relation to the patient’s clinical stability, and whether the patient was experiencing bleeding, vomiting, or diarrhea when the contact occurred. Sixty-seven (39%) health care–associated contacts occurred in settings where the patient was clinically stable and without bleeding, vomiting, or diarrhea; among these contacts, 45 (67%) were classified as high-risk on the basis of one or more PPE omissions (i.e., of gown, gloves, eye protection, or face mask); these persons were permitted to continue working in order to maintain local health care capacity. Five of the 67 contacts had direct skin-to-skin contact and one or more PPE omissions and were excluded from work until 21 days after their last exposure. At hospital C, the patient was clinically unstable, and health care providers were at risk for body fluid exposure. Among 68 identified contacts at hospital C, 25 (37%) were classified as high-risk and excluded from work through day 21 from their last exposure; 24 of these 25 contacts had one or more PPE omissions (i.e., gown, gloves, eye protection, or N95 respirator), and one had body fluid contact.

Laboratorians were evaluated on the basis of activities performed and the use of appropriate PPE; 34 contacts were identified across six laboratories; 23 (68%) of these were classified as high-risk, and were excluded from work for 21 days, on the basis of published recommendations for biosafety in microbiological and biomedical laboratories (*5*).

All 105 high-risk contacts without contraindications were offered oral ribavirin postexposure prophylaxis (*4*); however, most felt that their exposure did not warrant prophylaxis. Five (5%) contacts began prophylaxis, four stopped because of adverse reactions (e.g., nausea), and one completed the 10-day course. Among 158 monitored contacts, 43 (27%) reported any signs or symptoms, including five whose signs or symptoms were potentially consistent with Lassa fever; these persons were transported to an assessment or treatment hospital** under VHF precautions for evaluation and testing; all test results were negative.

## Preliminary Conclusions and Actions

The occurrence of this Lassa fever case and the ensuing public health response underscore the importance of eliciting a travel history from febrile patients, maintaining awareness of high-consequence infectious disease risk, and facilitating close coordination between clinical and public health partners. Well-developed VHF response planning and rapid test turnaround were essential to preventing transmission despite multiple possible exposures to this patient with fatal disease.

## Footnotes

*
https://www.cdc.gov/lassa-fever/about/index.html

†
https://www.cdc.gov/viral-hemorrhagic-fevers/php/public-health-strategy/people-with-suspected-or-confirmed-vhf-or-high-risk.html

§ Weakness or fatigue, fever, headache, chills, muscle aches, diarrhea, vomiting, unexplained bleeding or bruising, rash, chest pain, sore throat, cough, or dark urine.

¶ Risk was based on the patient’s clinical stability and caregivers’ use or omission of PPE. Anyone who provided care when the patient was unstable (rapid clinical deterioration, obtundation, or requirement for intubation or vasopressors) would have had to wear gown, gloves, eye protection, and an N95 respirator; omission of any of these would result in classification of the contact as high risk. https://www.cdc.gov/viral-hemorrhagic-fevers/hcp/guidance/ppe-clinically-stable-puis.html; https://www.cdc.gov/viral-hemorrhagic-fevers/hcp/guidance/ppe-clinically-unstable.html

** The Iowa Bureau of Preparedness and Response program has used funding from the Hospital Preparedness Program (an Administration for Strategic Preparedness and Response grant) and the Public Health Emergency Preparedness program (a CDC grant) to create a robust Highly Infectious Diseases system comprising four EMS transport agencies, two Level 3 assessment hospitals, and one Level 2 treatment center. This system was established in 2014 during the Ebola virus disease outbreak and has been continuously refined since then.
