# *Erratum:* Vol. 74, No. 11

**DOI:** 10.15585/mmwr.mm7413a4

**Published:** 2025-04-17

The report, “Notes from the Field: Response to a Case of Travel-Associated Lassa Fever — Iowa, October–November 2024,” contained two errors. 

On page 194, the sixth sentence of the Introduction should have read, “A blood specimen collected at hospital C was tested within hours at the Nebraska Public Health Laboratory using the **BioFire Global Fever Special Pathogens Panel** (*2*), which returned a presumptive positive result for Lassa virus.”

On page 196, reference 2 in the References should have read, “BioFire Defense. **BioFire Global Fever Special Pathogens Panel**. Salt Lake City, UT: BioFire Defense; 2025. https://www.biofiredefense.com/gfspecialpathogens/.”

